# SHP-2 in Lymphocytes' Cytokine and Inhibitory Receptor Signaling

**DOI:** 10.3389/fimmu.2019.02468

**Published:** 2019-10-25

**Authors:** Charlène Niogret, Walter Birchmeier, Greta Guarda

**Affiliations:** ^1^Department of Biochemistry, University of Lausanne, Épalinges, Switzerland; ^2^Max-Delbrueck-Center for Molecular Medicine (MDC) in the Helmholtz Society, Berlin, Germany; ^3^Institute for Research in Biomedicine, Università della Svizzera italiana, Bellinzona, Switzerland

**Keywords:** SHP-2 phosphatase, SHP-2 inhibitors, PTPN11 gene, lymphocytes, cytokine, inhibitory receptors of lymphocytes, PD-1, cancer

## Abstract

Somewhat counterintuitively, the tyrosine phosphatase SHP-2 (SH2 domain-containing protein tyrosine phosphatase-2) is crucial for the activation of extracellular signal-regulated kinase (ERK) downstream of various growth factor receptors, thereby exerting essential developmental functions. This phosphatase also deploys proto-oncogenic functions and specific inhibitors have recently been developed. With respect to the immune system, the role of SHP-2 in the signaling of cytokines relevant for myelopoiesis and myeloid malignancies has been intensively studied. The function of this phosphatase downstream of cytokines important for lymphocytes is less understood, though multiple lines of evidence suggest its importance. In addition, SHP-2 has been proposed to mediate the suppressive effects of inhibitory receptors (IRs) that sustain a dysfunctional state in anticancer T cells. Molecules involved in IR signaling are of potential pharmaceutical interest as blockade of these inhibitory circuits leads to remarkable clinical benefit. Here, we discuss the dichotomy in the functions ascribed to SHP-2 downstream of cytokine receptors and IRs, with a focus on T and NK lymphocytes. Further, we highlight the importance of broadening our understanding of SHP-2′s relevance in lymphocytes, an essential step to inform on side effects and unanticipated benefits of its therapeutic blockade.

## Introduction

Protein phosphorylation is a post-translational modification fundamental for intracellular signaling cascades and is therefore tightly regulated by kinases and phosphatases. SHP-2 (SH2 domain-containing protein tyrosine phosphatase-2, encoded by the *PTPN11* gene) is a broadly expressed, cytoplasmic phosphatase highly relevant for human health (1–4). In fact, *PTPN11* mutations cause the polymalformative Noonan and LEOPARD syndromes, two developmental disorders characterized by manifestations such as craniofacial abnormalities, growth defects, cardiac malformations, and—in some cases—mental retardation (5, 6). To understand the biological function of SHP-2, genetic mouse models have been generated. Full-body deletion of Shp-2 resulted in embryonic lethality due to multiple defects in mesoderm patterning (7), whereas inducible Shp-2 deletion in adult mice led to death within 6–8 weeks and was accompanied by bone marrow aplasia and anemia (8). Further, conditional Shp-2 deletion revealed the role of this phosphatase in the development of various organs and tissues, including in the nervous system, the heart, the mammary gland, the kidney, and the intestine (8–14). In most instances, the effects of SHP-2 have been ascribed to its positive function in regulating extracellular signal-regulated kinase (ERK) signaling downstream of a number of growth factor receptors (1–4). Overactivation of SHP-2 is also involved in multiple cancers, a notion that encouraged the development of small molecule inhibitors (2, 15–20). As discussed later, SHP-2 blockade markedly suppressed cancer growth in preclinical models and specific inhibitors are currently tested in clinical studies (19, 21–26).

In this review, we focus on the role of SHP-2 in T and natural killer (NK) lymphocytes, which are crucial players in immunity and in anticancer immunotherapy. Regrettably, the role of SHP-2 in these immune subsets remains incompletely understood. Whereas, SHP-2's function in activating ERK downstream of multiple growth factors has been firmly established, it is less well-characterized downstream of cytokines relevant for lymphoid cells. Further, a role for this phosphatase in “immune checkpoint” signaling cascades has been reported. Here, we discuss recent advances in the understanding of how SHP-2 shapes these pathways and highlight open questions that—with the advent of inhibitors for clinical use—are becoming increasingly pressing.

## Molecular Function of SHP-2

**Figure 1:**
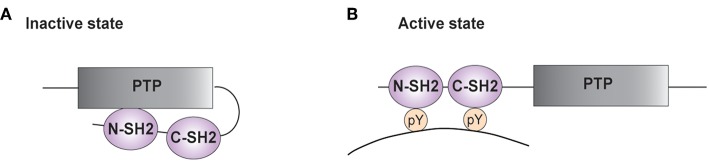
Structure of SHP-2. **(A,B)** A schematic representation of the phosphatase SHP-2 (SH2 domain-containing protein tyrosine phosphatase-2) is illustrated. The functional domains of SHP-2 comprise two SH2 domains [N-terminal SH2 (N-SH2) and C-terminal SH2 (C-SH2)] and a protein tyrosine phosphatase (PTP) domain. **(A)** In the absence of a tyrosine-phosphorylated substrate, the N-SH2 domain interacts with the PTP domain and blocks the catalytic site. **(B)** Interaction of SH2 domains with tyrosine-phosphorylated (pY) residues on targets enables phosphatase activity.

SHP-2 possesses two N-terminal SH2 domains (N-SH2 and C-SH2) and a central protein tyrosine phosphatase (PTP) core (Figure 1) (3, 4, 27–30). The PTP domain is highly conserved among classical PTP phosphatases and is responsible for the catalytic activity of these enzymes. It is characterized by the [I/V]HCSXGXGR[S/T] sequence, with the invariant cysteine being responsible for the nucleophilic attack of the phosphate group to be removed (31, 32). The C-terminal tail of SHP-2 contains tyrosine residues that can become phosphorylated and modulate the phosphatase activity (3).

In the inactive state, the N-SH2 domain interacts with the PTP region, limiting access of substrates into the active site (Figure 1A) (33–35). The auto-inhibition is relieved upon SH2 binding to phosphotyrosine residues on targets (Figure 1B). The importance of this autoinhibitory mechanism is confirmed by studies on the mutations of *PTPN11* associated to LEOPARD and Noonan Syndromes. The latter genetic disorder is caused by *PTPN11* gain of function mutations, whereas the clinically similar LEOPARD Syndrome is linked to mutations reducing the catalytic activity of SHP-2. Recent findings started unraveling this paradox, showing that mutations found in LEOPARD Syndrome, besides decreasing the phosphatase activity, affect the intramolecular interaction between the N-SH2 and the PTP domain, favoring the transition to its active conformation and producing a gain of function-like phenotype (36, 37).

**Figure 2:**
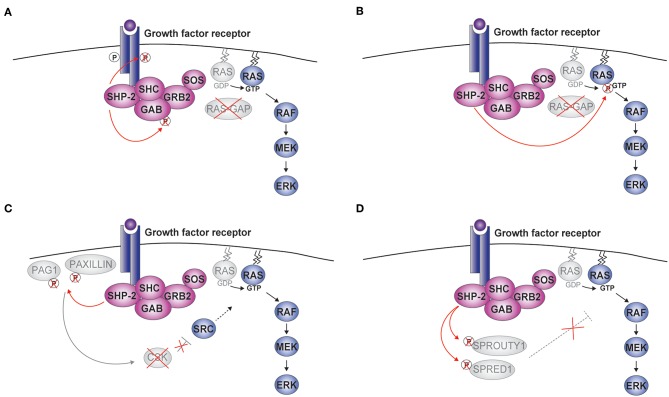
SHP-2-mediated activation of ERK. Upon cytokine binding, a complex including SHP-2, growth factor receptor-bound protein 2 (GRB2), and Son of Sevenless (SOS) is formed at the receptor. Four molecular mechanisms linking the phosphatase activity of SHP-2 to the activation of the RAS-RAF-MEK (mitogen-activated protein kinase kinase or MAPKK)-extracellular signal-regulated kinase (ERK) pathway are schematically illustrated **(A–D)**. CSK, c-src tyrosine kinase; GAB, GRB2-associated-binding protein; PAG1, phosphoprotein associated with glycosphingolipid microdomains; RAS-GAP, RAS-GTPase activating protein; SHC, Src homology 2 domain containing; SPRED1, Sprouty-related ena/vasodilator-stimulated phosphoprotein homology 1 domain-containing protein1.

Through the interaction of the SH2 domains with phosphotyrosine residues on targets, SHP-2 is recruited to various receptors, directly or indirectly through docking proteins such as Insulin Receptor Substrate 1 (IRS1) and GRB2-associated-binding protein 1 or 2 (GAB1/2) (Figure 2) (3, 38, 39). Upon recruitment, SHP-2 is found in a signaling complex comprising growth factor receptor-bound protein 2 (GRB2) and the associated Son of Sevenless (SOS) (38, 40–43). By promoting the conversion of RAS-bound GDP to GTP, SOS activates the mitogen-activated protein kinase (MAPK) pathway involving RAF-MEK (mitogen-activated protein kinase kinase or MAPKK)-ERK. The expression of a catalytically-inactive SHP-2 and the use of specific inhibitors demonstrated the importance of the phosphatase activity for ERK activation (16, 25, 44–46). Thus, SHP-2 is an atypical phosphatase involved in positively regulating intracellular signaling pathways through its catalytic function.

To explain how the phosphatase activity of SHP-2 stimulates the RAS-ERK pathway, five mechanisms have been proposed. First, SHP-2 was shown to dephosphorylate specific positions of the receptor (e.g., PDGFR) or GAB thus preventing the recruitment of the RAS-GTPase activating protein RAS-GAP (Figure 2A) (47–51). Opposite to SOS, RAS-GAP terminates the activation of the MAPK signaling pathway by inducing hydrolysis of RAS-bound GTP. Second, RAS tyrosine phosphorylation at position 32 negatively impacts on downstream signaling, possibly by favoring the interaction with RAS-GAP; by removing this modification, SHP-2 promotes ERK activation (Figure 2B) (52). Third, SHP-2 was found to eliminate phosphorylated docking sites on the scaffolding proteins Paxillin (PXN) and PAG1 (phosphoprotein associated with glycosphingolipid microdomains 1) (Figure 2C). These phosphorylation sites are involved in recruiting/modulating the activity of CSK (c-src tyrosine kinase), which suppresses receptor tyrosine kinase (RTK)-activated Src kinases and, indirectly, ERK signaling (53, 54). Fourth, Sprouty (SPRY) 1 and SPRED1 (Sprouty-related ena/vasodilator-stimulated phosphoprotein homology 1-domain-containing protein1) are known to inhibit ERK signaling and have been proposed to do so by multiple mechanisms acting at the level, downstream, or upstream of RAS (55). Interestingly, the function of SPRY1 and SPRED1 requires specific phosphorylations, which can be removed by SHP-2 (Figure 2D) (13, 56–58). Finally, two recent publications support a model whereby SHP-2's catalytic function is necessary for the assembly of the complex including SHP-2 itself, GAB, and GRB2 at the receptor. This model is attractive, as it suggests that the action of SHP-2 might involve more general mechanisms than interfering with specific inhibitory proteins. However, the underlying molecular events remain to be defined and might integrate the mechanisms described above (25, 59).

**Figure 3:**
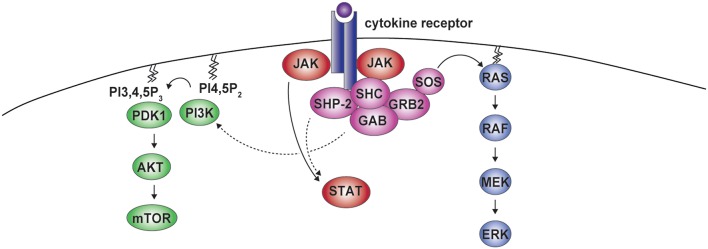
SHP-2 in cytokine receptor signaling. Cytokine binding to the receptor induces formation of the SHP-2-containing complex. Besides being involved in the activation of ERK, SHP-2 can modulate phosphoinositide 3-kinase (PI3K) activity. PI3K mediates the conversion of phosphatidylinositol 4,5-bisphosphate (PI4,5P_2_) into phosphatidylinositol 3,4,5-trisphosphate (PI3,4,5P_3_), which leads to phosphoinositide-dependent kinase 1 (PDK1) recruitment, AKT phosphorylation, and mammalian target of rapamycin (mTOR) activation. Residues phosphorylated by janus kinases (JAKs) in the cytoplasmic portion of the receptor act as binding sites for signal transducers and activators of transcription (STAT) proteins, that are further phosphorylated by JAKs, allowing dimerization and nuclear translocation. This pathway can also be modulated by SHP-2.

In addition to the ERK cascade, SHP-2 has been involved in the Phosphoinositide 3-kinase (PI3K)-AKT pathway. The adaptor GAB has been found to associate with SHP-2 and the PI3K p85 regulatory subunit, indirectly modulating PI3K signaling in response to selected cytokines (Figure 3) (38, 60–64). However, studies assessing PI3K/AKT activity or the phosphorylation of AKT at position 308, which is controlled by the PI3K-phosphoinositide-dependent kinase 1 (PDK1) axis (65), show negative as well as positive roles for SHP-2 on this pathway. For example, insulin- and epidermal growth factor (EGF)-dependent PI3K activation were found to be negatively influenced by SHP-2, most likely through the dephosphorylation of the p85 binding sites on the adaptor proteins GAB or IRS1 (64, 66–68). Conversely, SHP-2 interaction with p85 has been shown to be required for the association of the PI3K catalytic subunit p110 and for full PI3K activity downstream of insulin-like growth factor 1(IGF-1) (69). Similar effects were observed downstream of additional growth factors including insulin, PDGF, and granulocyte-macrophage colony-stimulating factor (GM-CSF) (63, 69–72). We therefore lack a unified view on the effects of SHP-2 on the PI3K pathway.

Along the same lines, SHP-2 has been reported to modulate the phosphorylation of signal transducers and activators of transcription (STAT) transcription factors downstream of various cytokines (Figure 3) (73). Upon engagement, cytokine receptors initiate signaling through Janus kinases (JAKs), which phosphorylate multiple residues in their cytoplasmic portions forming docking sites for STATs, that are themselves phosphorylated by JAKs to translocate to the nucleus and exert central transcriptional functions (73). On the one hand, SHP-2 was found to promote the dephosphorylation of different STATs, including downstream of interleukin (IL)-3, leukemia inhibitory factor (LIF), or IL-10 in cells of various origin (73–76). On the other hand, no effect or even the opposite outcome has been observed, as for instance in response to transforming growth factor-β (9, 73, 77–79). SHP-2 acts therefore downstream of several receptors to activate the ERK pathway and can modulate PI3K-AKT and JAK-STAT axes.

## Small Molecular Weight Inhibitors of SHP-2 for Cancer Treatment

Several cancers rely on overactive MAPK signaling. Indeed, activating mutations of *PTPN11* have been identified in juvenile myelomonocytic leukemia (JMML) (2, 20, 80–82). In many other cancer types, enhanced MAPK signaling is achieved through alternative mechanisms, such as alterations of RTKs like EGFR. Despite mutation of SHP-2 in tumors, particularly in solid ones, is an infrequent event, its key role in RTK-triggered signaling cascades renders it an attractive target for pharmacological intervention.

The identification of small molecule inhibitors for SHP-2 has however been a challenging endeavor, and no SHP-2 inhibitor has yet reached advanced stages of clinical trials (19, 83). A SHP-2 inhibitor, PHPS1, has been identified early and further developed into GS493 (17, 84). GS493 acts on purified SHP-2 in the nanomolar range, and was shown to inhibit breast cancer upon administration in mice (21). This and other SHP-2 inhibitors bind to or close to the active site of the enzyme. This straightforward approach is however potentially complicated by the high degree of homology across PTP catalytic domains, in particular with respect to Src Homology 2 (SH2) domain-containing tyrosine phosphatase 1 (SHP-1), the closest homolog of SHP-2 (85–87). More recently, inhibitors of SHP-2 have been reported, which act by new allosteric mechanisms (16, 25, 88). Two such compounds are SHP099, which stabilizes the inactive conformation of SHP-2 by occupying a tunnel-like binding site between the two SH2 and the PTP domain, and RMC-4550, which inhibits by a similar mode of action. SHP099 blocks SHP-2 in the nanomolar range, whereas RMC-4550 acts at even lower doses, with an IC_50_ of 0.58 nM. Both drugs were shown to limit the growth of xenografted cancers driven by oncogenic mutations of the RAF kinase and RAS member BRAF and KRAS, respectively (16, 22–26). Taken together, these data indicate that SHP-2 inhibition can be of use as a monotherapy.

However, cancer drug resistance is a massive clinical problem (89). Tumor cells often evade inhibition of proteins targeted by molecular therapies by re-activation of the signaling pathways via elaborate feedback mechanisms. This is the case for KRAS- or BRAF-driven cancers treated with MEK and BRAF inhibitors. The phosphatase SHP-2, being a crucial component in the signal transduction cascade between growth factor receptors and these downstream pathways, is an excellent potential target to battle drug resistance mediated by such cascades. This principle has been shown to work for BRAF inhibitor-resistant BRAF-mutant colon cancers (90). Treatment with BRAF inhibitor concomitant with genetic ablation or pharmacological inhibition of SHP-2 by the inhibitor GS493 prevented re-activation of MAPK signaling by feedback activation of the EGF receptor, inducing synthetic lethality of the transformed cells. In addition, in MEK inhibitor-resistant KRAS-mutant pancreatic, lung epithelial, and gastric cancer cell lines, simultaneous blocking of MEK and SHP2 acted synergistically, substantially hindering cell proliferation *in vitro* and tumor growth in xenograft models (23–26). Importantly, the combination treatment was well-tolerated, as evidenced by the similar body mass these mice maintained over time compared to vehicle-treated animals. Collectively, these works have provided proof-of-principle that small molecule inhibitors of SHP-2 can prevent resistance to MAPK pathway-targeting drugs in BRAF and KRAS mutant tumor cells. Together, these results establish SHP-2 blockade as a potentially powerful option to treat inhibitor refractory tumors in human patients.

Whereas SHP-2 is a central node in the commonly altered RTK/MAPK pathways, this phosphatase is mutated in few cancers, such as JMML (2). Nearly half of patients with SHP-2-mutated cancers bear strongly activating mutations that are thought to perturb its autoinhibited conformation, such as the common mutation of the position D61 and E76 in the N-SH2 domain. As the currently available allosteric inhibitors interact simultaneously with the C-SH2, N-SH2, and PTP domains, it is uncertain that successful suppression of such SHP-2 mutants is achievable in a clinical setting. This encourages further investigation to develop inhibitors targeting the catalytic site or the most common mutants, which might find broader application in patients with activating SHP-2 mutations (16, 25, 26, 91).

## SHP-2 in Cytokines' Signaling in T and NK Lymphocytes

*PTPN11* mutations found in JMML confer increased sensitivity to the growth factors GM-CSF and IL-3 (2, 15, 20, 92). These two cytokines share the β subunit of the receptor, which is common also to the receptor for IL-5, a cytokine important for the B cell and the eosinophil lineages. Upon cytokine stimulation, this receptor subunit recruits SHP-2, leading to the activation of the MAPK pathway and the interaction with the p85 subunit of PI3K (93–98). Lending support to the role of SHP-2 in these signaling cascades, a recent study demonstrated that Shp-2-deficient eosinophils failed to induce ERK activation upon IL-5 exposure, exhibiting reduced airway hyper-responsiveness in allergic models (99). Further to its role in pathways favoring myelogenous leukemias and normal myelopoiesis, SHP-2 is involved in the signaling by cytokines promoting hematopoiesis more broadly. Its function in the maintenance of hematopoietic stem cells and lineage progenitors has been attributed to the signaling downstream of multiple growth factors including stem cell factor (SCF), thrombopoietin (TPO), Fms-like tyrosine kinase 3 ligand (FLT3L), and interleukin (IL)-3 (8, 94, 95, 100–104). SHP-2 has also been implicated downstream of the receptors for cytokines important in mature immune cells, including lymphocytes (8).

Two decades ago, SHP-2 has been found to participate in the signaling induced by IL-6, a pleiotropic cytokine regulating inflammation, B cell responses, and T cell differentiation (105). An interaction between SHP-2 and the IL-6 receptor (IL-6R) subunit gp130 has been reported and mutation of the SHP-2 recruitment site suggested that this phosphatase was important to engage ERK and dampen STAT3 activation, limiting autoimmunity (106–113). Later studies showed that the same gp130 binding site recruited the JAK inhibitor Suppressor of cytokine signaling 3 (SOCS3), attributing to the latter the antagonism with STAT3, and confounding the role of SHP-2 (113, 114). These data indicate that, whereas SHP-2's function in activating the ERK pathway is widely accepted, the mechanisms underlying its effects on STAT activation shall be carefully evaluated. Therefore, the function of SHP-2 downstream of IL-6R and other less characterized gp130-containing receptors, such as the ones of IL-11, LIF, oncostatin M (OSM), and IL-27, which is of great relevance for T cells, await further experimental investigation.

SHP-2 has also been implicated in the response to IL-2 and IL-15 (60–62, 115–117). IL-2 is essential for regulatory, effector CD4^+^, and effector CD8^+^ T cells. IL-15 is important for the survival of memory CD8^+^ T cells and for development, survival, and activation of NK cells, two cytotoxic subsets which are central to immunity against intracellular pathogens and cancers. The receptors for IL-2 and IL-15 share the γ_c_ and the CD122 subunits (also known as IL-2 receptor β subunit). Phosphorylation of SHP-2, a phenomenon occurring upon receptor recruitment, was found to be largely dependent on the latter receptor subunit (62, 116). In agreement with what has been observed for other growth factor receptors, IL-2 and IL-15 stimulation led to the formation of a complex comprising SHP-2, GAB2, GRB2, and the PI3K p85 subunit (60, 61). Downstream of the IL-2R in T cells, SHP-2 has been involved in ERK engagement, while no or a positive effect was observed on STAT5 activation (79, 117, 118). Recently, we investigated the role of Shp-2 downstream of IL-15 stimulation in primary murine NK cells. While STAT5 phosphorylation was largely unaffected, Shp-2 was essential for ERK engagement (78). Interestingly, genetic ablation of Shp-2 also impaired phosphorylation of AKT (position 308), metabolic raise, and NK cell expansion in response to IL-15, suggesting a significant connection to cell metabolism (78). The family of γ_c_-dependent cytokines comprises other members, which are instrumental for the lymphocytic compartment and have receptor subunits different from CD122. While stimulation with IL-4 and IL-7 did not induce phosphorylation of GAB2 or SHP-2 itself, IL-21 and the related thymic stromal lymphopoietin (TSLP) were shown to engage ERK and AKT and lead to phosphorylation of SHP-2 or other components typical of the SHP-2-containing complex (119–121). These results reveal therefore a role for SHP-2 in regulating the response to several cytokines and suggest a broader involvement, encouraging future studies.

## SHP-2 and Inhibitory Receptor Signaling in T and NK Cells

SHP-2 is considered a central molecule downstream of inhibitory receptors (IRs). IRs are expressed by immune cells and regulate their function in diverse contexts. The cytoplasmic portion of IRs contains inhibitory motifs, such as immunoreceptor tyrosine-based inhibition motifs (ITIMs) and tyrosine-based switch motifs (ITSMs). Both motifs bear tyrosine residues that are phosphorylated upon IR engagement and recruit SH2 domain-containing phosphatases to antagonize activating cascades (122). During NK cell development, specific IRs interact with major histocompatibility complex (MHC) class I molecules, preventing a state of anergy (123–127). This process known as “NK cell education” mainly depends on SHP-1, the closest homolog of SHP-2. Biochemical evidence demonstrated SHP-1 recruitment to the ITIMs in the cytoplasmic portion of these IR, whereas elegant genetic approaches showed its essential function in maintaining NK cell responsiveness (126, 127). Notably, SHP-2 has also been shown to interact with NK cell IRs, suggesting a role in these suppressive signals (123, 124, 128). Through *in vivo* genetic approaches, we could however rule out a major role for this phosphatase in this pathway (78).

On T cells, transient IR expression is observed upon T cell receptor (TCR) triggering. Instead, constitutive IR display is associated and contributes to a dysfunctional state—known as “exhaustion”—that impairs T cell proliferative and effector capacities in cases of chronic antigen exposure (129–131). In particular, T cell exhaustion has been described in the context of chronic infections and cancer. Blockade of IR-mediated inhibitory circuits has recently transformed cancer immunotherapy, enabling to reactivate anti-tumoral T cell responses, and better control disease (131–133). Therefore, it is important to define the molecular events mediating IR effects, which might represent novel targets for pharmacological intervention.

Earlier studies showed interaction of SHP-2 with the intracellular tail of IRs and this correlated with the inhibition of T cell activation pathways (134–141). For instance, SHP-2 has been shown to interact with the cytoplasmic tail of the IR B- and T-lymphocyte attenuator (BTLA), whose blockade shows promise in immunogenic cancer treatment (142–144). Further, one of the most relevant IR is programmed cell death 1 (PD-1), whose blockade reinvigorates T cells against various cancer types. Its engagement has been shown to affect both TCR and co-stimulatory signaling (136, 141, 145). SHP-2 has been reported to robustly interact with the cytoplasmic tail of PD-1 and to exert a negative effect on interleukin (IL)-2 production, a surrogate read-out for TCR signaling (135, 138, 146, 147). This was observed in T cell hybridomas and in the Jurkat T cell line upon TCR and PD-1 engagement (138, 146). Of note, one of the effects of PD-1 engagement is the impairment of ERK activation (136, 138, 146, 148). The possibility that SHP-2 inhibits this cascade downstream of PD-1 is difficult to reconcile with its well-documented role in promoting it downstream of growth factor receptors. Moreover, despite the role of SHP-2 in TCR signaling remains controversial (79, 149–157), a positive effect on ERK engagement has been observed also in this context (149, 152, 153). The dichotomy in the effects of SHP-2 could be explained by a model in which IRs reduce the availability of the phosphatase, thus preventing its contribution to the ERK cascade, or by a very distinct regulation of SHP-2 activity downstream of IRs and growth factor receptors.

To evaluate whether the absence of SHP-2 reverted T cell exhaustion in more physiological conditions, we generated mice lacking this phosphatase in T cells. In the context of chronic viral infection, we found that antiviral *Ptpn11*-knockout T cells presented typical signs of exhaustion, exhibiting compromised cytokine production and tolerable immunopathology (156, 158, 159). In addition, immunogenic cancers developed in these mice with kinetics similar to the ones observed in the control groups (156). Along these lines, studies by others showed that the growth of immunogenic tumors in mice lacking Shp-2 in T cells was moderately retarded or accelerated, but even in the former case the effects on tumor growth were distant from the ones of PD-1-deficiency (154, 160, 161). One interpretation is that the therapeutic effects of PD-1 blockade are not largely mediated by T cells, a quite unlikely hypothesis in light of cytotoxic T cell depletion results (131, 162). With respect to this question, conditional PD-1 deletion will be informative. Most importantly, genetic deletion or pharmacological inhibition of Shp-2 did not prevent the therapeutic benefit of antibody-mediated PD-1 blockade (156, 161). These results challenge the possibility that IRs antagonize TCR and co-stimulatory signaling by reducing the availability of this phosphatase and imply that PD-1 signaling occurs in the absence of Shp-2 activity.

**Figure 4:**
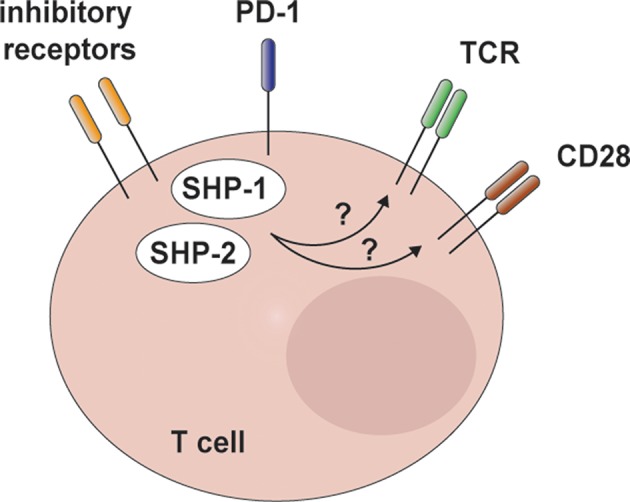
SHP-2 and redundant mechanisms in T cell inhibitory receptor signaling. Recent *in vitro* data indicate that SHP-2 and SHP-1 are engaged by programmed cell death 1 (PD-1) and possibly other inhibitory receptors involved in T cell exhaustion. Notably, these two phosphatases exert redundant functions in limiting T cell receptor (TCR)/CD28 signaling and interleukin-2 (IL-2) production in Jurkat T cells.

Intriguingly, SHP-2 has been shown to dephosphorylate the cytoplasmic tail of PD-1 as part of a feedback loop (138, 141), even suggesting a role in the termination of the inhibitory function of this IR and the possible accumulation of other SH-containing proteins and phosphatases in its absence. In addition to SHP-2, SHP-1 has been shown to interact with PD-1 and other IRs, albeit to a lesser extent (136, 137, 143, 144). Given their homology, a recent study explored the possibility that these two phosphatases exert redundant functions in PD-1 signaling. This work showed how only the abrogation of both phosphatases robustly relieved the inhibitory effects of PD-1 on TCR- and CD28-induced signaling, including ERK, in Jurkat T cells (Figure 4) (147). This important study paves the road to evaluate SHP-1 and SHP-2 redundancy in anticancer T cells *in vivo*.

## Discussion

On the one hand, detailing IR signaling in exhausted T cells is of high clinical value. Yet, the lack of definite knowledge on the molecular events downstream of IRs delays the design of small molecule inhibitor-based interventions. Better understanding the mechanism of action of SHP-2 in these cascades is therefore relevant and timely. On the other hand, our understanding of SHP-2 function downstream of important growth factor receptors remains incomplete from a mechanistic viewpoint and in immune cells, lymphocytes in particular. Investigation in this direction would help answer the long-standing question of how a phosphatase enhances selected signaling and suggest novel targets for immunomodulation. Furthermore, currently available genetic models allow detailing the physiological contribution of SHP-2 *in vivo* with unprecedented accuracy. In the future, the study of tissue-specific and inducible knockout mice will be essential to define the immune subset-specific functions of SHP-2, while limiting the confounding effects of compensatory mechanisms.

Preclinical and clinical studies assessing the efficacy of SHP-2 inhibitors in cancer therapies raise the question on possible side effects, and immune cells shall be carefully examined in this respect. We deem that studies mapping SHP-2's functions are a prerequisite for evaluating these aspects, which will be highly relevant if immunotherapeutic approaches would be used in complement to SHP-2 inhibitors. Besides, these investigations might suggest unanticipated benefits of SHP-2 inhibitor therapies, as for instance in normalizing deregulated immune responses, such as in autoimmunity, and atopy.

## Author Contributions

CN, WB, and GG wrote the manuscript.

### Conflict of Interest

Unrelated projects in GG laboratory are supported by OM Pharma (Vifor Pharma) and Novartis Foundation. CN is now a clinical affairs specialist at Bio-Rad laboratories and works on unrelated projects. The remaining author declares that the research was conducted in the absence of any commercial or financial relationships that could be construed as a potential conflict of interest.
